# Diverging ideas of health? Comparing the basis of health ratings across gender, age, and country

**DOI:** 10.1016/j.socscimed.2020.112913

**Published:** 2020-12

**Authors:** Patrick Lazarevič, Martina Brandt

**Affiliations:** a Austrian Academy of Sciences, Vienna Institute of Demography, Vordere Zollamtsstraße 3, 1030, Vienna, Austria; b TU Dortmund, Institute for Sociology, Emil-Figge-Str. 50, 44227, Dortmund, Germany

**Keywords:** Self-rated health, Survey of Health, Ageing and Retirement in Europe (SHARE), Cross-national comparison, Epidemiology, Response behavior, Measurement invariance, Europe

## Abstract

**Background:**

Self-rated health (SRH) is arguably the most widely used generic health measurement in survey research. However, SRH remains a black box for researchers. In our paper, we want to gain a better understanding of SRH by identifying its determinants, quantifying the contribution of different health domains to explain SRH, and by exploring the moderating role of gender, age groups, and the country of residence.

**Method:**

Using data from 61,365 participants of the fifth wave (2013) of the Survey of Health, Ageing and Retirement in Europe (SHARE) living in fifteen European countries, we explain SRH via linear regression models. The independent variables are grouped into five health domains: functioning, diseases, pain, mental health, and behavior. Via dominance analysis, we focus on their individual contribution to explaining SRH and compare these contributions across gender, three age groups, and fifteen European countries.

**Results:**

Our model explains SRH rather well (R^2^ = .51 for females/.48 for males) with functioning contributing most to the appraisal (.20/.18). Diseases were the second most relevant health dimension (.14/.16) followed by pain (.08/.07) and mental health (.07/.06). Health behavior (.02/.01) was less relevant for health ratings. This ranking held true for almost all countries with only little variance overall. A comparison of age groups indicated that the contribution of diseases and behavior to SRH decreased over the life-course while the contribution of functioning to R^2^ increased.

**Conclusion:**

Our paper demonstrates that SRH is largely based on diverse health information with functioning and diseases being most important. However, there is still room for idiosyncrasies or even bias.

## Highlights

• Self-rated health (SRH) is explained via five dimensions of health information.
• Amount of explained variance by dimension is compared by gender, age group, and country.
• Functioning and diseases were the most important dimensions.
• Only minor differences in importance were found by gender and country.
• Importance of diseases/behavior decreased with age, increased for functioning.

## 1 Introduction

Self-rated health (SRH) is the most used generic health indicator in a wide array of scientific disciplines. It is usually collected via a single question asking for the respondents' health rating on a four or five-point scale that is typically labeled either with asymmetrical (excellent to poor; ‘US-version’) or symmetrical (very good to very bad; ‘WHO-version’) response options in survey research (Jylhä, 2009). In many studies, SRH is seen and treated as a valid generic health measurement and is used both as a resource (e.g., as a restriction or prerequisite for social participation) and as an outcome (e.g., when researching preservation or improvement of health). Its usage is most commonly (if at all) justified with its repeatedly demonstrated relation to mortality and it can be seen as an inclusive, dynamic, health behavior affecting, and resource reflecting measure of health status (Benyamini, 2011; Idler and Benyamini, 1997).

However, due to the vagueness of the question, survey respondents are relatively free to decide what to base their health rating on. The consequence is that researchers cannot be sure as to what exactly is measured by SRH (Garbarski, 2016; Jylhä, 2009). This uncertainty is especially striking as SRH is routinely used to measure health inequalities and their relation to, e.g., socio-economic aspects (see, e.g., a meta analysis on SRH and income-inequality by Kondo et al., 2009). Consequently, research on the health determinants of SRH is necessary to better understand what respondents base their appraisal on.

So far, studies examining the health-related determinants of SRH found that it is also strongly and consistently correlated to a wide array of other common health indicators like symptoms or diagnoses of diseases and especially pain (e.g., Tornstam, 1975), (consequences of) risky health-behaviors like being under-/overweight/obese (e.g., Manderbacka et al., 1999) or smoking (e.g., Wang and Arah, 2015), mental health issues such as depression (e.g., Kivinen et al., 1998), or health-related restrictions of the functional status or the daily life (e.g., Suchman et al., 1958). Even if there is a vast amount of studies on determinants of subjective health-ratings, this should not hide the fact this research oftentimes lacks an underlying theoretical model, and does rarely touch upon the relative importance or weight of the identified determinants for the rating and/or the potential issue of measurement equivalence across different subgroups of the population, e.g., gender, age groups, as well as across different countries. Yet, all these aspects are highly relevant for using SRH in empirical research.

Firstly, research on and with SRH without an underlying theoretical model of the response process suitable to guide the analysis risks resulting in fragmented and isolated findings that do not contribute to scientific progress or purposeful evidence-based policy. Secondly, empirically determining to which extent respondents base their health-ratings on which health domains is important for survey research not least in order to know whether SRH is a suitable indicator of the intended concept of health in a given analysis. Thirdly, if there are group differences in the health concept used to judge one's health status, any comparisons of SRH across these groups are called into question. This problem of measurement equivalence or differential item functioning, which is known as ‘response shift’ in the context of age differences (Sprangers and Schwartz, 1999), country-specific response styles in the context of international differences (Jürges, 2007), or simply as sex/gender differences when referring to differences between male and female respondents (Schulz et al., 1994), can either produce health differences when there are none or it can obscure actual group differences. The purpose of this paper is to (1) provide a cognitive model of the response process for SRH to guide concrete research for a better and more systematic understanding of SRH and (2) to contribute to research regarding SRH's underlying determinants, their relative importance, and possible group differences in order to lead to a better understanding of this generic health indicator in comparative settings.

Therefore, we firstly develop and describe a general cognitive model of the response process for SRH based on existing models on the cognitive process of answering survey questions and models of the process of health ratings and relevant empirical research. From this general model we then derive a more specific analytical model for our analysis of the relative contributions of different health domains to SRH and potential group differences. In this context, we are the first to systematize the determinants of SRH into different health domains (the theoretical discussion of this paper can also be found in greater detail elsewhere (Lazarevič, 2018; Lazarevič, 2019)). This model is followed by a short review of the literature concerning these strands of research. We then describe the data and methods used for our empirical analysis and the results from a linear regression explaining SRH by five types of health indicators already mentioned above: functioning, diseases, pain, mental health, and behavior. The results are presented separately by gender in order to compare women and men. We then analyze the contributions of these five aspects to *R*^2^ for three age groups and 15 European countries.

## 2 Theoretical models: the cognitive process and our analytical model

### 2.1 The cognitive process of health-ratings

**Fig. 1:**
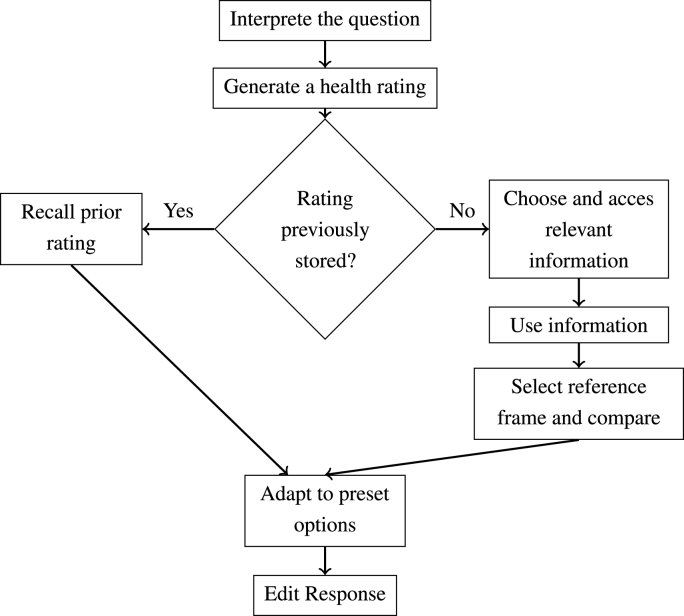
Cognitive model to explain the process of health-ratings.

As a theoretical background for our analysis we have synthesized a model (Fig. 1) by combining the general cognitive model of the response process for survey questions by Tourangeau (1984) in its extended form by Strack and Martin (1987) with the response process of SRH as proposed by Knäuper and Turner (2003) and Jylhä (2009). The model comprises, in accordance with the response process described by Strack and Martin (1987), four major steps and every step potentially influences the subsequent steps.

The *first step* is the comprehension and interpretation of the question. The respondent has to interpret what is meant by ‘(general/overall) health’. Respondents might be particularly influenced by preceding questions if they are also concerned with health aspects in the form of assimilation or contrast/subtraction effects (Garbarski, 2016). In both cases, strong survey design effects are to be expected: In the first case this would mean that respondents more or less summarize the information they have already given (Garbarski et al., 2015) while the latter would mean that respondents evaluate their health aside from the health information they already provided (Tourangeau et al., 1991).

After interpreting the question, as a *second step*, respondents need to generate an opinion. For this, they can either recall an evaluation of their health from memory (e.g., in panel surveys or if they are frequently occupied with thinking about their health) or they can generate a health evaluation on the spot. In the latter case they first have to choose which (health) information is relevant for their rating. They might consider factors like medical diagnoses, observations about their functional status, pain experiences, and body perceptions as indicated by Knäuper and Turner (2003) and Jylhä (2009). However, due to a changing public awareness of mental health and in line with the 1948 definition of health by the World Health Organization (2006), one might also consider depression or depressive symptoms as relevant factors (Han and Jylhä, 2006; Kivinen et al., 1998; Schnittker, 2005). Information regarding these factors has to be recalled from the respondents memory implying that more salient information, e.g., acute health problems or pain (Knäuper and Turner, 2003), is more likely to be recalled and deemed relevant because it is more accessible. The respondents then incorporate the available information into one global evaluation of health by either weighting the recalled factors in some way in order to incorporate them (Anderson, 1971) or by using simpler heuristics like focusing on the most available/salient information (Tversky and Kahneman, 1973). Either way it can be assumed that the information at hand is incorporated somewhat systematically, nonetheless, the systematics can vary between different groups of respondents or even individually. As a last part of this step, respondents select a reference frame (e.g., age-peers or themselves at an earlier point in time) and compare their overall health to it (Cheng et al., 2007; Knäuper and Turner, 2003; Krause and Jay, 1994; Strack and Martin, 1987).

Once respondents generated an overall evaluation of health, the *third step* of rating their health lies in choosing the most adequate response option. Obviously, this step is strongly influenced by questionnaire features (Schwarz, 1999) like response options (Lee, 2015) or their order (Garbarski et al., 2015), implying problems of comparability especially between surveys that implement SRH differently. Lastly, as the *fourth step*, respondents may choose to edit their answer due to factors like social desirability (Strack and Martin, 1987), e.g., in order to not seem frail or to gain sympathy (Maddox, 1962).

Of course, it is likely that the process and outcome of each step are modified by personal characteristics like belonging to certain demographic or socio-economic groups. In this paper, we explicitly focus on rather stable demographic factors (i.e., gender, age, and the country of residence). We chose to leave aside other possible group differences such as education or income as they are more prone to change during the life-course and strongly influence not only response behavior but also the base of health ratings, i.e., their health. Gender might play a role because women and men might have different concepts of health or find different health domains more (or less) relevant than others, e.g., due to gender specific health-reporting norms (Caroli and Weber-Baghdiguian, 2016; Undén and Elofsson, 2006; Zajacova et al., 2017). The same presumably applies to age due to older respondents' greater experience or even (perceived) normativeness of (adapting to) chronic diseases, co-morbidity, health-related limitations, and general physical and cognitive decline – both individually and in age-peers. This perception potentially influences how older respondents interpret the meaning of health, which information is most salient, or how they incorporate the available information in manifold ways, e.g., changing health aspirations or standards, adaptation processes, or susceptibility to methodological context effects (Idler, 1993; Knäuper et al., 2007; Krause and Jay, 1994; Maddox, 1962; Simon et al., 2005; Sprangers and Schwartz, 1999; Spuling et al., 2017; Tornstam, 1975). Further, the country of residence captures a complex blend of the respondent's cultural background, welfare state, and language of the interview that might all influence what weight is placed on which health domains or which answer is chosen for reasons such as a varying access to (health) care, a different interpretation of the question or value labels used, or (culturally based) country-specific response styles (Bardage et al., 2005; Jürges, 2007; Viruell-Fuentes et al., 2011).

### 2.2 Analytical model

**Fig. 2:**
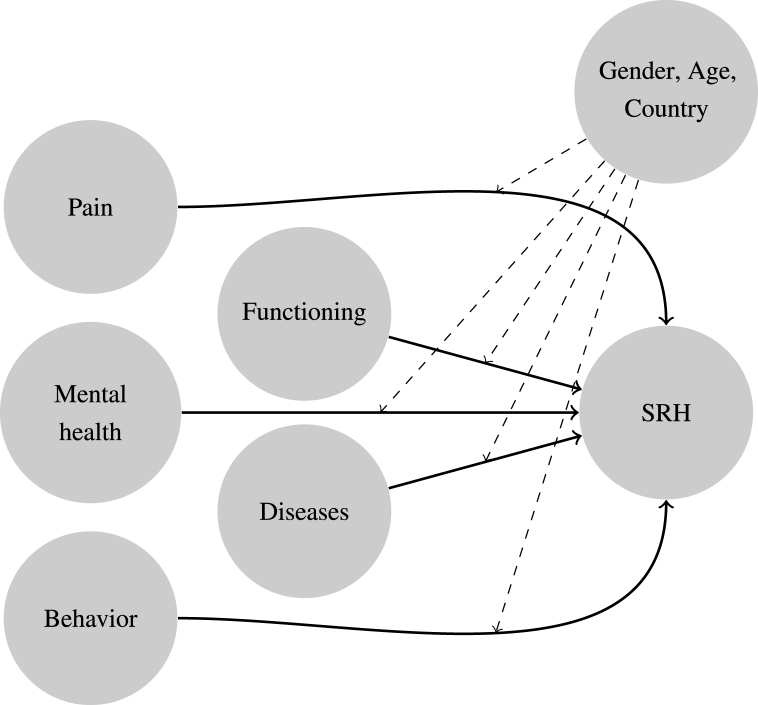
Analytical model for explaining SRH by health dimension.

To enable an empirical test of parts of the general cognitive model of the response process, we further developed an analytical model that mainly focuses on the second step, i.e., generating a health rating. A depiction of this model can be seen in Fig. 2. This model states, in line with the model of the cognitive process, that respondents choose and recall knowledge pertinent to their health from memory and incorporate it into one overall rating. For a greater clarity and comparability, we assign all health information to five general types or domains: functioning, diseases, pain, mental health, and behavior.

(Physical) *functioning* represents how well the respondents function in their daily lives or how limited they are, respectively, as well as their general fitness (e.g., Barsky et al., 1992; Benyamini et al., 1999; Jylhä et al., 1998; Leinonen, 2002; Leinonen et al., 1999; Liang et al., 2007; Nakano, 2014; Pinquart, 2001; Quinn et al., 1999; Schulz et al., 1994; Shooshtari et al., 2007; Simon et al., 2005; Suchman et al., 1958). This can, e.g., be measured via self-reports of limitations or via performance tests. The aspect *diseases* serves as a general category for all (chronic) diseases and health conditions that can be diagnosed and are known to the respondent (e.g., Cott et al., 1999; Fylkesnes and Førde, 1991, 1992; Goldberg et al., 2001; Jylhä et al., 1998; Kivinen et al., 1998; Leinonen, 2002; Leinonen et al., 1999; Mellner and Lundberg, 2003; Nakano, 2014; Quinn et al., 1999; Schulz et al., 1994; Segovia et al., 1989; Shooshtari et al., 2007; Simon et al., 2005; Singh-Manoux et al., 2006; Tornstam, 1975). *Pain* and its intensity is classified here separately since it is especially salient to the respondents and can not necessarily be attributed to a specific health problem (e.g., Cott et al., 1999; Idler, 1993; Shooshtari et al., 2007; Tornstam, 1975). The category of *mental health* comprises all issues connected to mental health, especially depressive symptoms, diagnosed depression, or intake of medication against depression or anxiety as an additional objective signal for the respondents (e.g., Goldberg et al., 2001; Han and Jylhä, 2006; Kivinen et al., 1998; Leinonen, 2002; Leinonen et al., 1999; Nakano, 2014; Pinquart, 2001; Quinn et al., 1999; Schnittker, 2005; Spuling et al., 2015). Lastly, *behavior* is an additional category representing behaviors that are known to the respondents to have adverse health effects such as smoking or being overweight (e.g., Cotter and Lachman, 2010; Imai et al., 2008; Manderbacka et al., 1999; Månsson and Merlo, 2001; Noh et al., 2017; Tang et al., 2017; Wang and Arah, 2015; Zajacova and Burgard, 2010). These might be taken by the respondent as an indicator of their health status even if they do not explicitly affect their health status yet.

The incorporation of information on these five domains is, however, likely modified by aspects like gender, age, and country. Reasons can, for example, lay in group-specific health-reporting norms, health aspirations, reference frames, or their culture (e.g., how to view or talk about health and illness) or simply the language. This aspect represents both the potential differential choosing of reference frames and group-specific ways to evaluate one's health and is, in our analysis, accounted for by using separate models for each subgroup.

## 3 Previous studies: relative importance and group differences

### 3.1 The relative importance of health indicators

Even though there are a lot of studies investigating the influence of health indicators on SRH in one way or another, systematic approaches to examine the relative importance of indicators or health domains are fairly scarce, especially in recent years and in relation to their relevance in terms of understanding what is measured. Yet, evidence from this line of research is vital for working with SRH in order to know what it actually measures. The first study examining this subject matter was conducted by Tornstam (1975). In his paper he found that aches and serious diseases were the most relevant aspects when explaining SRH. Barsky et al. (1992) however, studying hospital patients, found only restrictions of the functional status as a significant health-related determinant of SRH with somatization and hypochondriasis being much more important. Quinn et al. (1999) found in their article that physical health, comprising both functioning and diseases, is more important for SRH than mental health while Ratner et al. (1998) found only physical health to be relevant for SRH. Shooshtari et al. (2007) found with Canadian data from the National Population Health Survey (NPHS) that while all aspects considered in the present paper (i.e., functioning, diseases, pain, mental health, and behavior) are relevant for rating one's health, functioning and diseases were the most important.

One of the few studies that explicitly sought to explore the relative importance of health indicators on SRH was conducted by Singh-Manoux et al. (2006). They used data from the Whitehall II and Gazel cohort study in order to quantify the contribution of different aspects of health towards the explanation of SRH. Their conclusion was that health indicators such as symptoms, longstanding illnesses, health problems, and mobility accounted for 35–41 percent of SRH's variance, depending on the data set, while other aspects were less relevant. Using rather broad categories of variables, they also showed that physical health was the most important health domain, followed by mental health, and health behavior.

### 3.2 Differences in how groups of respondents rate their health

There are not many studies on group differences in health ratings yet, even though systematic differences might be expected, e.g., in terms of gender, age, and country with gender being the most prominently researched in the pertinent literature. For example, men might put greater weight on physical functioning while women signify the importance of the absence of illnesses (Peersman et al., 2012) or only women's assessment might be influenced by depressive symptoms (Leinonen et al., 1999). However, some studies did not find any notable differences in the rating behavior by men and women (Jylhä et al., 1998; Undén and Elofsson, 2006; Zajacova et al., 2017), complicating clear predictions about the results to be expected.

A second aspect that might be relevant for how respondents rate their health might be their age since the aspirational level of health decreases with age, meaning that older respondents potentially have lower expectations or are more tolerant of health problems than younger respondents (Tornstam, 1975) or have different frames or reference, e.g., specific health problems, physical functioning, or health behaviors (Krause and Jay, 1994). If, e.g., older persons do not take medical conditions or diseases into account when evaluating their health in a survey interview (while younger respondents do), their responses are not directly comparable regarding health. The same applies to other groups like men and women or respondents from different countries. If they would base their appraisal on different sets of indicators, weight them differently, or generally show different response behaviors, researchers could not directly compare their health measurements. The already cited study by Tornstam (1975), for example, found overall weaker negative effects of adverse health due to lower health aspirations in older age. Other studies found differences in the relevance of various health domains, such as: symptoms and mental health being more relevant for young-old (51–55) while old-old (71–75) lay more weight on chronic diseases (Jylhä et al., 1986); mental health being more relevant for older respondents while for younger respondents physical health/functioning and chronic diseases were more important (Pinquart, 2001; Schnittker, 2005; Jylhä et al., 2001); younger people in general using more diverse aspects in rating their health (Shooshtari et al., 2007); the importance of mental health being stable while medical conditions and functioning losing in importance with age (French et al., 2012); chronic conditions being stable and mental health being more relevant in younger cohorts (Spuling et al., 2015); and behavior being less relevant for SRH in older age (Manderbacka et al., 1999).

The third aspect possibly responsible for different rating behaviors explored in this paper is the country of residence, reflecting different aspects like culture, language, and welfare state regimes. Despite this complex mixture of factors, the pertinent literature did not find evidence for differential rating behavior with European data (Jylhä et al., 1998; Bardage et al., 2005; Verropoulou, 2009).

This short overview illustrates the dire need for more studies aimed at systematically quantifying and comparing the relative importance of different health domains commonly associated with SRH across different groups. While we can expect functioning and diseases, as classical determinants of subjective health, to be of great importance for SRH, the role of pain, mental health/depression, and behavior is rather unclear. The same, maybe to an even greater extent, is true for group differences in rating behavior as some studies came to contradicting results. The state of research for all three aspects (i.e., gender, age, and country) is inconclusive regarding the type of effect to be expected (age) or whether to expect any meaningful differences at all (gender and country). This ambiguity further demonstrates the necessity of research on this subject.

## 4 Data and method

### 4.1 Data

For our analysis we use data of the 5th wave of the Survey of Health, Ageing and Retirement in Europe (SHARE) from 2015 comprising a wide array of health information for more than 61,000 respondents from 15 European countries (Austria, Belgium, Czech Republic, Denmark, Estonia, France, Germany, Italy, Israel, Luxembourg, Netherlands, Slovenia, Spain, Sweden, Switzerland) aged 50 years or older. The multivariate analyses comprise information from 61,365 respondents (33,796 women and 27,569 men).

### 4.2 Analysis and measurement

In order to implement the analysis according to our analytical model, we used linear regression models with SRH as the dependent and all aforementioned health-related variables as independent variables. As *R*^2^ in linear regressions quantifies the variance explained by independent variables (i.e., health information), it indicates to what extent SRH is systematically determined by health information known to the respondent, thus providing an empirical benchmark of our analytical model and the criterion validity of SRH.

The independent variables were blocked according to the proposed five types of health information: functioning, diseases, pain, mental health, and behavior:

**Self-rated health** As a measure for SRH, we used the question ‘Would you say your health … ?’ with US-version response options (i.e., ‘Excellent, Very good, Good, Fair, Poor’). We treated SRH quasi-metric which enables a linear regression analysis. This approach can be justified both by the very low skewness of this variable's distribution (approximately .24 for women and 0.16 for men) as well other studies demonstrating linear relationships between SRH and other health indicators (Perruccio et al., 2012). This approach was also corroborated by regression diagnostics (i.e., linear relationships, no problems with multicollinearity or outliers). Additionally, we replicated all analyses with generalized ordinal logit regression models (Williams, 2006), which came to the same results. In SHARE, SRH is not preceded by any other health-specific question, implying a free interpretation of the meaning of health by the respondents.

**Functioning** In order to operationalize (physical) functioning, we used five different aspects comprising both self-reports and physical performance tests. As for the latter, we used both a measure of grip strength and the chair stand (Cooper et al., 2011) to explain SRH. To this end, we generated two dummy-variables that represent being in the lowest performance quartile of one's own gender (weakest for grip strength, slowest for chair stand) and not having a measurement taken. Item-nonresponse for these variables can be seen as informative nonresponse since it can be assumed that it means respondents were (deemed or feeling) unfit to participate in the measurement (Herzog and Rodgers, 1992). Since this would mean that the item-nonresponse is missing not at random (MNAR), a simple exclusion would bias the results (Gardette et al., 2007). As for the self-reports, we used count variables for the number of restrictions in (instrumental) activities of daily living ((I)ADL; 13 items, e.g., *dressing, including putting on shoes and socks* or *shopping for groceries*) and mobility (10 items, e.g., *walking 100 m* or *stooping, kneeling, or crouching*) and a global question regarding the presence of functional limitations (i.e., the global activity limitation indicator (GALI)). In order to account for the nonlinear association of SRH and the two count variables, we transformed them utilizing an inverse hyperbolic sine transformation (log(*x*_*i*_ + (*x*_*i*_^2^ + 1).^5^)). This transformation is similar to a logarithmic transformation but allows the transformation of zero-values (Burbidge et al., 1988; Zhang et al., 2000), which are common for these (I)ADL and mobility restrictions.

**Diseases** Diseases were operationalized via a count variable of different health conditions and diseases diagnosed by a doctor as reported by the respondent (17 diagnoses like *high blood cholesterol* or *cancer* and including *other conditions, not yet mentioned*) and a general question whether or not the respondent suffered from a chronic or long-term health problem. The count variable was also transformed via an inverse hyperbolic sine transformation. Substituting this variable with individual variables for each condition did not increase explanatory power of the model.

**Pain** In order to measure pain, we included a single general question whether or not the respondent was troubled with pain at the time of the interview. For respondents experiencing pain, this question was supplemented with information on whether they consider the pain to be mild, moderate, or severe.

**Mental health** Mental health was measured through the number of depressive symptoms on the Euro-D scale (Prince et al., 1999) which was also transformed like the other count variables. Additionally, we included a general question regarding taking medication against depression or anxiety in our model as a proxy for diagnoses in this regard.

**Behavior** The measurement of behavior was twofold to depict two common types of risky health behavior or its consequences, respectively: smoking and non-normal weight. Smoking was captured with a question whether the respondent currently smokes while the body-mass-index (BMI) was calculated as the self-reported weight (in kg) of the respondent divided by their squared self-reported height (in m). To account for the nonlinear relationship of BMI and health, we used dummy-variables for being underweight, overweight, and being adipose. We explicitly did not include alcohol consumption and physical activity, which are typically seen as health-related behaviors, in this health domain. While both behaviors are (subjective) reasons for bad health and therefore fit this health domain, they are also strongly restricted by it (i.e., not being able to drink alcohol or exercise due to medication or functional limitations) resulting in an overestimation of the relevance of this health domain.

We firstly applied the general model to men and women separately, while also quantifying the contribution to *R*^2^ by each of the five health domains. A discussion of these results will be followed by a figure showing the contribution of the five types of health information by gender and age group in order to examine age-differences in how European respondents rate their health. Lastly, we compare the relative amount of explained variance for each of the 15 analyzed European countries to demonstrate the extent of country-specific health ratings and thus the comparability of self-rated health across countries.

In order to assess the health domains’ contributions to *R*^*2*^, we conducted dominance analyses with the Stata-module domin (Luchman, 2013). This approach compares *R*^2^ for all possible subsets of variables or variable-sets in order to determine the variance explained by them or, in other words, their contribution to overall *R*^2^ (Budescu, 1993; Luchman, 2014, 2015). If there are differences in the importance of dimensions between genders, age groups, or respondents from different countries, they are reflected in these analyses accordingly. To compare these contributions, we estimated confidence intervals through bootstrapping (10,000 samples for each model). We use these confidence intervals as a criterion for substantial differences in the importance of health dimensions for SRH between and within groups.

## 5 Results

Table 1 shows the regression results separately by gender. The models, comprising extensive health information, explained 51 percent of SRH's variance for women and 48 percent for men, documenting that SRH is heavily reliant on the health information known by the respondent and potentially ascertainable in surveys. This supports its use as a simple and inclusive measure of generic health representing a host of health indicators. It can be seen that all measured health indicators significantly influenced SRH for women in the expected direction. The same was true for men with the exception that being troubled by mild pain and being underweight did not significantly influence them in their health ratings when controlling for other health-related factors. All coefficients except for these two variables were rather similar in size between genders. Further, a missing measurement on each performance test was significantly related to SRH, suggesting informative nonresponse.

**Table 1:** Results from OLS-regressions explaining self-rated health (b-coefficients and amount of explained variance by dimension).

	Women	Men
**Functioning**	*19.96%*	*18.44%*
Global activity limitation indicator	−0.34***	−0.38***
Number of restrictions in daily life^a^	−0.07***	−0.04*
Number of restrictions in mobility^a^	−0.11***	−0.13***
*Grip strength (*ref*: middle 50%)*		
No measurement	−0.11***	−0.16***
Stronger 25%	0.10***	0.11***
Weaker 25%	−0.08***	−0.10***
*Chair stand (*ref*: middle 50%)*		
No measurement	−0.21***	−0.17***
Faster 25%	0.11***	0.08**
Slower 25%	−0.12***	−0.05*
**Diseases**	*14.39%*	*15.52%*
Chronic diseases (ref: none)	−0.23***	−0.32***
Number of diseases^a^	−0.23***	−0.25***
**Pain (RC: none)**	*8.29%*	*6.57%*
Mild	−0.10***	−0.05
Moderate	−0.19***	−0.19***
Severe	−0.33***	−0.32***
**Mental Health**	*7.05%*	*6.38%*
Medication for depression (ref: no)	−0.13***	−0.09*
Number of depressive symptoms^a^	−0.16***	−0.16***
**Behavior**	*1.53%*	*0.87%*
BMI (ref: normal (18.5 ≤ BMI ≤ 25))		
Underweight (BMI < 18.5)	−0.18***	0.08
Overweight (25 ≤ BMI < 30)	−0.07***	−0.06**
Adipose (BMI ≥ 30)	−0.14***	−0.12***
Current smoker (ref: no)	−0.07**	−0.12***
Adj. R^2^	0.51	0.48
n	33,796	27,569

p ≤ 0.05 p ≤ 0.01 p ≤ 0.001.

a Inverse hyperbolic sine transformation to account for nonlinear relationship.

**Fig. 3:**
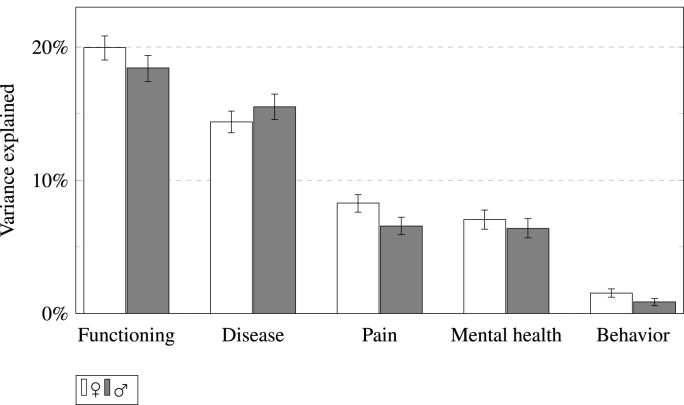
Amount of explained variance accounted for by health dimension by gender (95%-Confidence intervals).

As can be seen from Table 1 and Fig. 3 the most relevant health domains, functioning and diseases, together accounted for around a third of the variance of SRH for both men and women. These health domains were followed by pain with 8 percent and 7 percent respectively and then mental health with approximately 7 and 6 percent. The least relevant health domain in our analyses were behavioral variables with two percent for women and one percent for men. There was a notable difference between the explanatory power of functioning and diseases in that functioning explained more variance. Moreover, there was a slight gender difference in the amount of explained variance by pain and behavior which was greater for women than for men. Overall, however, we would argue that the gender differences, albeit observable, were rather small and that European women and men are remarkably similar in how they rate their health, replicating results already shown by Zajacova et al. (2017) with US data.

**Fig. 4:**
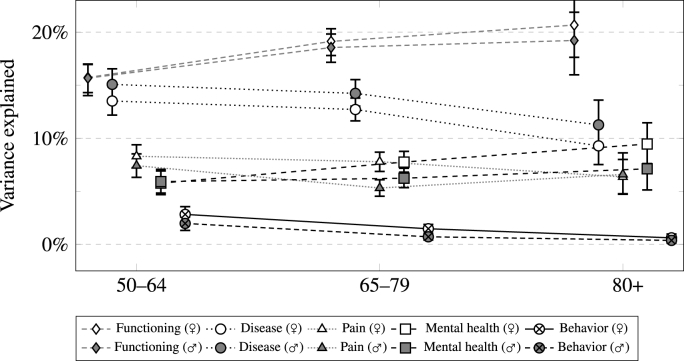
Amount of explained variance accounted for by health dimension by gender and age-group (95%-Confidence intervals).

A comparison of the three age groups separately by gender regarding the health domain's explained variance can be seen in Fig. 4. While there were, with the exception of pain in the middle age group, no meaningful differences between genders, it shows that there were clear and consistent differences in how the relevance of functioning, diseases, and behavior differs between age groups. Even though explained variance by functioning immensely increased over the three age groups, it decreased for the relevance of diseases and, to a lesser extent, behavior. Pain and mental health, however, were remarkably stable in their contribution to *R*^2^, with only a slight increase in relevance of mental health in women.

These results show differences as well as congruencies in how people from different age groups rate their health. It is also worth noting that there were, with a minor exception for pain in one age group, no marked gender differences in the importance of any health dimension. This corroborates the previous impression of similarities of rating behavior between genders overall. Generally, overall *R*^2^, as can be seen from Table 2, did not vary too much between age groups or genders.

**Table 2:** Adjusted R^2^ and number of cases for separate models by gender and age.

	Women			Men		
	50–64	65–79	80+	50–64	65–79	80+
Adj. R^2^	0.46	0.49	0.46	0.46	0.45	0.44
n	15,948	13,769	4,079	12,572	11,944	3,053

**Fig. 5:**
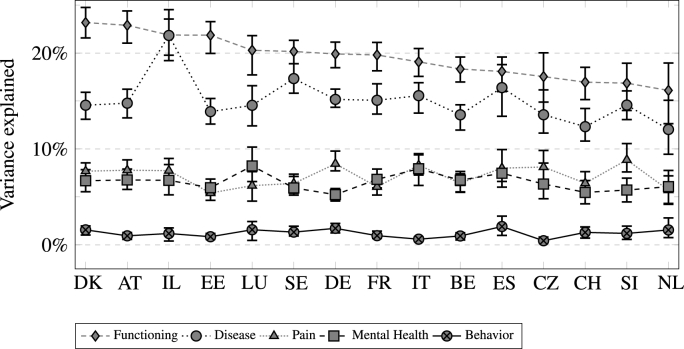
Amount of explained variance accounted for by health dimension by country (95%-Confidence intervals).

Fig. 5 shows the share of explained variance by the five types of health information for each European country used in our analysis. Since gender differences were relatively low in the previous analyses (and also in this case), only overall results are shown. The countries were sorted by the amount of variance explained by functioning. Overall, country differences appear rather small: The general ranking of importance, with functioning and diseases being most relevant, pain and then mental health in the middle, and behavior being the least relevant, held true for every single country in our analysis. Although there was some variation in overall *R*^2^ reported in Table 3, this variation did not seem to be systematic in any way.

**Table 3:** Adjusted R^2^ and number of cases for separate models by country.

	DK	AT	IL	EE	LU	SE	DE	FR	IT	BE	ES	CZ	CH	SI	NL
Adj. R^2^	0.53	0.53	0.59	0.48	0.50	0.51	0.50	0.48	0.51	0.46	0.52	0.46	0.42	0.47	0.41
n	3,879	3,957	2,112	5,284	1,536	4,352	5,452	4,298	4,455	5,286	5,793	5,253	2,943	2,753	4,012

Nevertheless, there were minor exceptions in which country-specific health rating behavior was observed in terms of contribution of different dimensions to overall *R*^2^. For over half of the countries (nine out of 15), functioning was more important than diseases, while the opposite was never the case. Additionally, there were only two countries (Germany and Slovenia) where pain was noticably more important than mental health. Notably, in all other countries there were no significant differences in the importance of functioning and disease, or pain and mental health respectively. This suggests an overall quite similar rating-process of general health in the analyzed countries.

## 6 Conclusion and discussion

The purpose of this paper was to identify, quantify, and compare the relevance of five different health domains – functioning, diseases, pain, mental health, and behavior – between different sociodemographic groups as to gain insight into what information is relevant to their health ratings. In order to do so, we analyzed data from the more than 61,000 respondents aged 50+ living in fifteen European countries collected in the 5th wave of the SHARE and compared the results between genders, age groups and countries of residence. The explanatory power of our models is relatively high since almost half of the variance of SRH was explainable with these health-related data. This finding corroborates early findings of “the centrality of objective health status in explaining self-assessments of health” (Maddox, 1962, p. 183). Apparently (and unsurprisingly), SRH is based to a large extent on health information known to the respondent. Still, it should be noted that also half of SRH's variance was not related to SHARE's rather comprehensive health data, leaving much room for differences due to health knowledge, non-health related idiosyncrasies, and even bias. Especially the influence of non health-related aspects, such as respondent or survey or interviewer characteristics, potentially biasing SRH merits further investigation.

Interestingly, missing values for performance tests turned out to be negatively related to overall SRH. This can be explained by the fact that missing performance tests are related to health because the interviewer or respondent deems the respondent in too bad health to participate in the test, thus creating nonresponse. This indicates that missingness for these variables is indeed MNAR and therefore excluding persons without measurement would presumably bias results of health-related research.

One main result of this paper is that functioning and diseases are by far the two most relevant health domains when it comes to rating one's health. Ranking by contribution to explaining SRH, they are followed by pain and mental health and then by behavior which appears to be only of subordinate importance for SRH-scores. As can be seen from our subgroup analyses and consistent with earlier research, there were no marked or systematic differences by gender (e.g., Jylhä et al., 1998; Undén and Elofsson, 2006; Zajacova et al., 2017) or (European) country (e.g., Jylhä et al., 1998; Bardage et al., 2005; Verropoulou, 2009). In this sense, the health ratings in these groups were rather congruent regarding to what information is used for health ratings. This suggests that comparing SRH between genders and (European) countries is somewhat unproblematic as they mostly rate their health in a similar way.

Yet, there were strong and systematic differences by age group in that functioning explained more of SRH's variance in older age groups while the opposite was true for both diseases and behavior. While younger cohorts of this 50+ population appear to base their health more strongly on diagnoses of diseases and health conditions, older respondents lay more weight on how well they function. Behavior, in general, appears to be only relevant for respondents younger than 80, which might also reflect selective mortality. This suggests that respondents of different ages indeed diverge in their understanding of what constitutes ‘health’ and how to rate it. However, these results might also reflect different challenges these age groups face, e.g., increasing functional limitations in older age. Nonetheless, this alternative explanation does not challenge the overarching finding that the contribution of functioning, diseases, and behavior to SRH systematically varies by age group. This confirms the existence of age-specific response behaviors as health information is differently reflected in this indicator by age. If the goal is to measure health in a consistent manner across age groups, these differences have to be taken into account. Nevertheless, our analysis was limited to European countries and respondents aged 50+ and as such cannot be generalized to other populations or contexts. Further research with other populations are advisable to attain a better understanding of the mechanisms underlying SRH.

These findings demonstrate that SRH measures generic health inconsistently across age groups in the sense that there are differences in the extent to which health domains are reflected in it. This can be seen as undesirable in a generic measure of health. However, as most surveys typically have very limited space for health measurement, the need for a short but robust collection of generic health information will likely persist. In order to harness the benefits of SRH, e.g., its inclusivity, it is desirable to find a way to curb its main disadvantage, i.e., a lack of consistency by age. One option to accomplish this can be seen in priming, i.e., standardizing the interpretation of ‘health’ by first asking about health aspects that are deemed relevant to generic health (Strack et al., 1988; Lee and Schwarz, 2014; Garbarski, 2016). Complementing SRH by combing it with factual information, e.g., via factor analytical models, could pose another way to achieve an improved short generic health measurement. The Minimum European Health Module (MEHM), which was designed by Robine et al. (2003), could provide a starting point for the study of both of these options as it comprises SRH and global questions regarding functional limitations and chronic diseases, i.e., the two most relevant health dimensions in this study. Further research on the feasibility and usefulness of these approaches promises significant improvements of the measurement of health in survey research.
